# Lissajous curves as aerial search patterns

**DOI:** 10.1038/s41598-024-60803-2

**Published:** 2024-05-15

**Authors:** J. Josiah Steckenrider, Mitchell Miller, Rory Blankenship, Victor Trujillo, James Bluman

**Affiliations:** 1 Department of Civil and Mechanical Engineering, United States Military Academy, West Point, NY USA; 2 Department of Mathematical Sciences, United States Military Academy, West Point, NY USA

**Keywords:** Unmanned aerial systems, Lissajous curves, Search, Path optimization, Numerical simulation, Predictive modeling, Aerospace engineering, Electrical and electronic engineering, Mechanical engineering, Applied mathematics, Computational science

## Abstract

Manned and unmanned systems are prevalent in a wide range of aerial searching applications. For aircraft whose trajectory is not or cannot be planned on-the-fly, optimal deterministic search pattern generation is a critical area of research. Lissajous curves have recently caught attention as excellent candidates for all kinds of aerial search applications, but little fundamental research has been done to understand how best to design Lissajous pattern (LP)s for this use. This paper examines the optimization of these search patterns from analytical, numerical, and data-driven perspectives to establish the state of the field in Lissajous curves for aerial search. From an analytical perspective, it was found that the average expected distance between a Lissajous searcher and a random target on a unit square approaches 0.586 as search time increases. Furthermore, an analytical approximation for the average searcher speed was found to guarantee error of no more than 22.1%. Important outcomes from the numerical optimization of Lissajous search patterns include the development of an intuitive evaluation criterion and the conclusion that irrational frequency ratios near 0.8 typically yield highest performance. Finally, while a robust predictive model for fast pattern optimization is yet out of reach, initial results indicate that such an approach shows promise.

## Introduction

### Background and motivation

The use of unmanned aerial systems (UAS) continues to proliferate, and thousands of new applications surface each year. The low cost of operating most UAS and the low barrier to entry has enabled operators to find uses for autonomous flying vehicles that are still relatively new and potentially disruptive to both commercial and government sectors. According to the International Civil Aviation Organization, there were over 2 million extant UAS in 2021 and this number is expected to rise to over 6.5 million in 2030^1^. UAS have been successfully incorporated into a host of activities including agriculture, aerial photography and videography, construction, mining, mapping and surveying, delivery services, and emergency response^2^.

**Figure 1:**
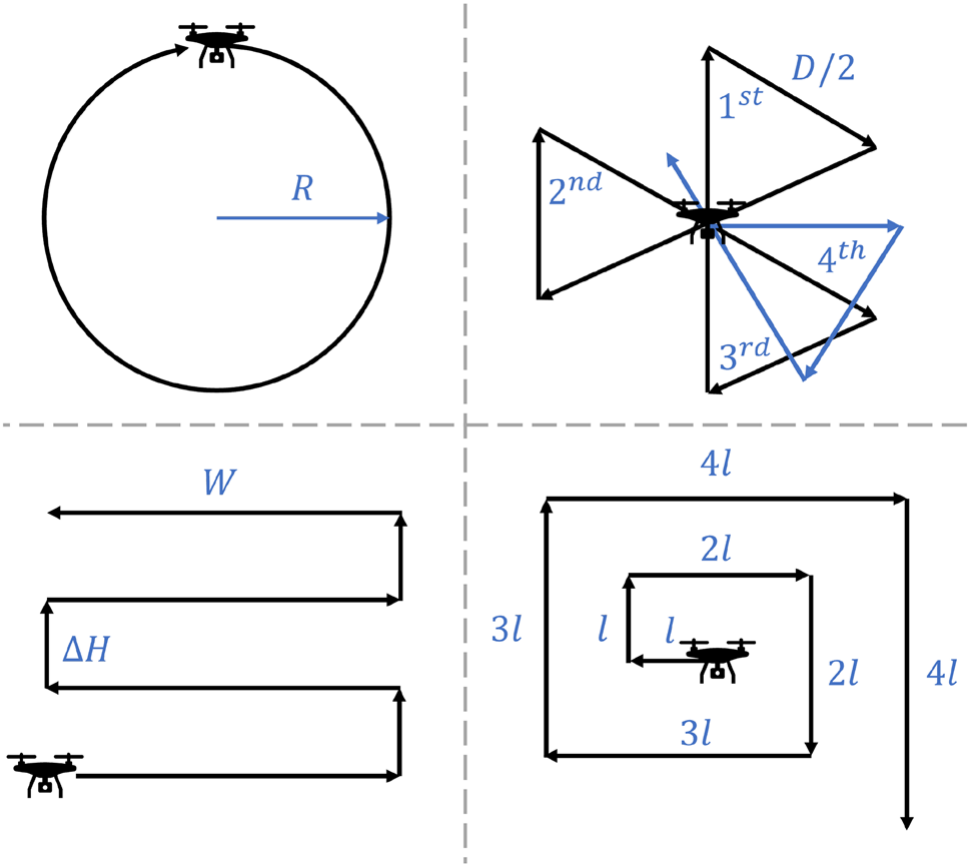
Four common deterministic search patterns prescribed in the International Aeronautical and Maritime Search and Rescue Manual^3^. From upper-left to lower-right: circle search, sector search, parallel track search, and expanding square search.

Utilizing UAS in search missions for emergency or disaster response is an obvious choice. Aerial vehicles have a particular advantage over grounded searchers since they can operate free from the restrictions of terrain and vegetation, and their sensors typically benefit from an elevated viewpoint. Search missions are often tedious and demanding, requiring significant endurance to be effective. Large UAS have much longer endurance than their comparable manned counterparts and can be fitted with state-of-the-art sensors and communication devices to aid in a search. Small UAS are also used increasingly in search and rescue missions due to their low cost and portability in scenarios as diverse as open ocean search^4^ to mountain avalanche scenarios^5^ to urban terrain^6,7^ to forested areas^8^. Additionally, as sensor technology continues to improve with higher resolution, lower weight, and lower power requirements, the benefits of using small UAS become increasingly amplified. Any means of leveraging the strengths of unmanned vehicles to make these missions more efficient and successful would be value-added.

Given the prevalence of UAS in technological solutions to modern search problems, research on how to most effectively implement aerial systems in such scenarios has become increasingly important. The solution space of a UAS for a given problem is largely dependent on the specific tasks at hand and the constraints imposed by the autonomous capabilities of the aerial system. The levels of UAS control and autonomy vary by platform and application. The National Institute of Standards and Technology (NIST) created the Autonomy Levels For Unmanned Systems (ALFUS) framework^9^ which defines Modes of Operation from remote control to fully autonomous^10^. Beer et al. reviewed decades of autonomy literature and proposed their own system of ten levels of autonomy with a focus on sensing, planning and acting^11^. This paper focuses on systems that would be considered autonomous or semiautonomous according to the NIST guidelines and level 7 (share control) or higher within Beer’s framework.

### Related work

Using UAS to assist in search and rescue has been an active area of research for over a decade. A key challenge for any autonomous or semi-autonomous system, especially in the context of aerial search, is path planning. Path planning can occur before or during missions, and can be updated in response to new information gained during the mission. Such information theoretic approaches to path planning are an area of active research^12,13^, and although adapting a path in light of information gained during search sounds attractive, there can be several practical drawbacks. One of the most significant hindrances in aerial search contexts is the excessive computational cost of continuously updating the search pattern, especially for small UAS with limited onboard computational assets and power. Moreover these patterns can be ineffective when little information about the search domain is known or made available.

Many recent ideas have been proposed in the areas of path planning as well as maintaining control of multiple unmanned aerial vehicles (UAV) flying simultaneously in support of a search mission. In 2009, Lin and Goodrich proposed an intelligent path planning method for wilderness search and rescue, however their technique relies heavily on a pre-determined probability distribution map to drive the performance of their search^14^. More recently Baker et al.^15^ introduced a coordinated Monte Carlo tree search algorithm which showed good performance against more basic search methods, but the method relies on knowledge of the situation on the ground prior to the start of the search. Although appropriate for post-disaster scenarios such as after an earthquake event, it is less well-suited to more dynamic scenarios.

Hayat et al. proposed a multi-objective optimization algorithm to allocate tasks and plan paths for a team of UAVs^16^. This work envisions a large search area where communications can only be assured through a mesh network of air vehicles which also utilize sensors to aid in their search. The flight paths are updated to maintain network integrity, but the path itself is not updated based on information from onboard sensors. San Juan and colleagues^17^ introduce a discrete path planning technique using four separate strategies, all of which assumes some level of prior knowledge about the location of the subject of the search based on a Risk/Occupancy Map, terrain, or other data. Research by Rahmes et al.^13^ focuses on creating a probability map which is updated at each last known position to calculate the next position for the UAS which maximizes the likelihood of locating the target in order to avoid UAS spending too much time searching in low-probability areas.

Echeveste et al.^12^ propose a UAS planning method with a focus on mapping the concentration of ground contamination by using Kriging variance to estimate the concentration in the entire area of interest based on five random sample points. In order to achieve the greatest reduction in uncertainty with each additional sample point, the study uses Variance Driven Sampling (VDS) to sample subsequent points with the greatest variance. However, this method is computationally expensive and may require the UAS to travel the across the entire search space to reach its next sample point.

Finally Xing et al.^18^ developed a multi-UAV cooperative system for search and rescue based on the 5th Generation You Only Look Once (YOLO) algorithm. Their framework includes the ability to free-graft multiple UAVs, independent control of each UAV, real-time target detection, and monocular positioning. Although sophisticated and able to accommodate heterogeneous search agents, the algorithm relies on hand selected initial flight paths and does not perform real-time updates to the path itself in spite of agent-to-agent communication with search status updates. There remains a lack of investigation into deterministic patterns that are both effective and efficient at finding targets in different scenarios.

**Figure 2:**
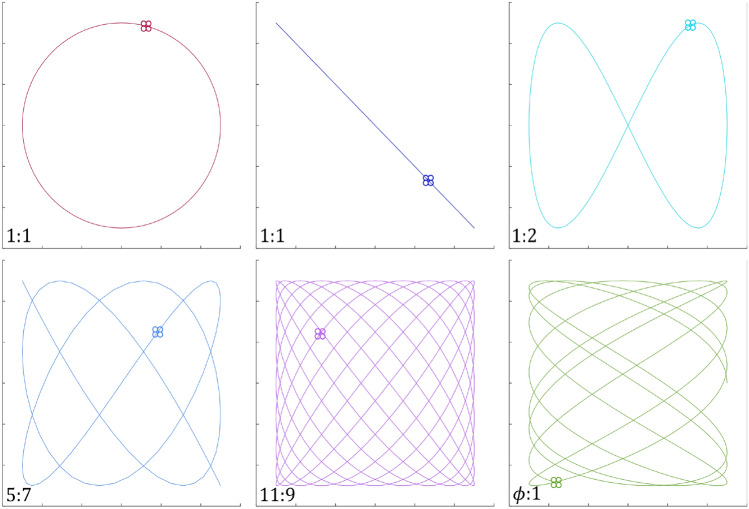
Assorted Lissajous curves. See section “Features of Lissajous curves” for a more detailed explanation.

Historically employed deterministic patterns are outlined by the International Aeronautical and Maritime Search and Rescue Manual^3^. Figure 1 shows examples of the most common patterns that have proven to be effective for maritime applications. In general, deterministic search patterns can often be classified as either cyclical or comprehensive. Cyclical patterns are usually designed for contexts where a moving target might be intercepted, and as such, they commonly do not cover an entire search space. In contrast, comprehensive patterns are designed to methodically search a whole area to guarantee the target will be located by the completion of the search. A weakness of comprehensive patterns is that they trade a potentially faster detection time for higher confidence of overall mission success. The hybrid pattern proposed in^19^ uses Lissajous curves to execute aerial search. This approach combines cross-domain sweeping motions with methodical area coverage to provide a thorough yet efficient interrogation of a search area. Search methods based on Lissajous curves can be both cyclical and comprehensive as demonstrated in Fig. 2, and they show significant promise as a potential alternative search method to those in current use^20^. In particular, LPs have been shown to more quickly reach an 80% confidence threshold of mission completion when optimized for assumed target size^21^ and are especially effective against canonical patterns when the target is modeled as adversarial^22^. Lissajous flight patterns can be quickly generated, are easily tuned to accommodate a wide range of scenarios, and do not require oversight or input about target location probabilities.

### Original contributions

The primary objective of this research is to comprehensively investigate the design of Lissajous search patterns for maximum efficacy from analytical, numerical, and data-driven perspectives. Original contributions include not only insights into optimal LP design for UAS search, but also a few mathematical derivations that are more generally useful in their own right. These include: (1) generalized continuous and discrete formulations for a Lissajous curve arbitrarily located and rotated in two dimensions, (2) the expected distance between a known searcher location and a randomly located target uniformly distributed on a unit square, (3) a symmetric linear approximation of the Pythagorean theorem, and (4) an approximate relationship between the average speed of a Lissajous curve and the parameters that describe it. Section “Lissajous curves” first establishes what Lissajous curves are, including a few existing formulations and some discussion about their features. Everything that follows is put forward as novel work: section “Analysis of LPS for aerial search” addresses LPs from a purely analytical perspective, motivating the need for numerical methods which are detailed in section “Numerical assessment and design of Lissajous search patterns”. Section “Predictive modeling for Lissajous search pattern design” investigates the use of predictive models for more efficient hyperparametric pattern design, and section “Conclusions and future applications” conveys concluding thoughts and future directions of the research.

## Traditional search patterns

While aerial vehicles can be controlled in a variety of ways, waypoint navigation is perhaps the most flexible and widely used. Waypoint navigation requires the generation of discrete locations in space which an agent must visit at specific times. The density of waypoints required to sufficiently construct a search pattern depends on the complexity and curvature of the pattern. The following subsections describe waypoint generation for the four canonical search patterns used almost exclusively in the field today, depicted in Fig. 1. It should be noted that all the patterns discussed here assume a constant searcher altitude. While three-dimensional search certainly has importance, we leave 3D considerations for future work.

### Circle search

Because circle search (CS) patterns can only be well approximated by many straight line segments, they may require well over ten waypoints to adequately construct. The *x* and *y* coordinates for a circular search agent are obtained by the following:

1 $$\begin{aligned} \begin{bmatrix} x_k \\ y_k \end{bmatrix} = R\begin{bmatrix} \cos {\Big (\frac{v}{R}k\Delta t\Big )} + {\bar{x}} \\ \sin {\Big (\frac{v}{R}k\Delta t\Big ) + {\bar{y}}} \end{bmatrix}, \end{aligned}$$

where *R* is the radius of the search circle, *v* is the velocity of the agent, $[{\bar{x}} \; {\bar{y}}]^\top$ are the coordinates of the center of the circle, and $\Delta t$ is the time the searcher takes to move between waypoints. This time step parameter controls the resolution of the pattern.

#### Sector search

The sector search (SS) path uses a cyclical, overlapping pattern whose waypoints are most easily defined by a recurrence relation. The $k^{th}$ *x* (east) and *y* (north) coordinates and two-dimensonal (2D) heading $\theta$ of a search agent are given by:

2 $$\begin{aligned} \begin{bmatrix} x_k \\ y_k \\ \theta _k \end{bmatrix} = \begin{bmatrix} x_{k-1} \\ y_{k-1} \\ \theta _{k-1} \end{bmatrix} + \begin{bmatrix} D_{k-1}\cos {\theta _{k-1}} \\ D_{k-1}\sin {\theta _{k-1}} \\ \frac{2\pi }{3} \end{bmatrix}, \end{aligned}$$

where

3 $$\begin{aligned} D_{k-1} = {\left\{ \begin{array}{ll} D, &{}k-1 \text { is even} \\ D/2, &{}k-1 \text { is odd} \end{array}\right. }, \end{aligned}$$

and *D* is a distance determined by the breadth of the search region. This pattern repeats when *k* is a multiple of 6, at which point an angular offset is often added.

#### Parallel track

The parallel track search (PTS) pattern methodically covers a rectangular search space by scanning the width or height of the space at regular offsets. The waypoints for a PTS moving from south to north can be generated as follows:

4 $$\begin{aligned} \begin{bmatrix} x_k \\ y_k \\ \theta _k \end{bmatrix} = \begin{bmatrix} x_{k-1} \\ y_{k-1} \\ \theta _{k-1} \end{bmatrix} + \begin{bmatrix} D_{k-1}\sin {\theta _{k-1}} \\ D_{k-1}\cos {\theta _{k-1}} \\ \frac{\pi }{2}\sqrt{2}\sin {\Big (\frac{\pi (k-1)}{2}-\frac{\pi }{4}\Big )} \end{bmatrix}, \end{aligned}$$

where

5 $$\begin{aligned} D_{k-1} = {\left\{ \begin{array}{ll} \Delta H, &{}k-1 \text { is even} \\ W, &{}k-1 \text { is odd} \end{array}\right. }, \end{aligned}$$

*W* is the width of the search region, and $\Delta H$ is the desired distance increment in the *y* direction.

#### Expanding square

The expanding square (ES) begins at or near the center of a search domain and moves outward along a spiraling path. The critical waypoints for such a pattern are given by:

6 $$\begin{aligned} \begin{bmatrix} x_k \\ y_k \\ \theta _k \end{bmatrix} = \begin{bmatrix} x_{k-1} \\ y_{k-1} \\ \theta _{k-1} \end{bmatrix} + \begin{bmatrix} l_{k-1}\cos {\theta _{k-1}} \\ l_{k-1}\sin {\theta _{k-1}} \\ \frac{\pi }{2} \end{bmatrix}, \end{aligned}$$

where

7 $$\begin{aligned} l_{k-1} = l\lfloor (k-1)/2\rfloor , \end{aligned}$$

and *l* is a distance parameter governed by the size of the search space.

## Lissajous curves

### Continuous parametric formulation

Despite their potential for complexity, Lissajous curves have a relatively simple mathematical formulation in two dimensions. Equation (8) give the parametric representation of these curves in the *x* and *y* directions:

8 $$\begin{aligned} \begin{bmatrix} X(t) \\ Y(t) \end{bmatrix} = \begin{bmatrix} A_{x}\sin {(\omega _{x}t+\phi _{x})} \\ A_{y}\sin {(\omega _{y}t+\phi _{y})} \end{bmatrix} \end{aligned}$$

where $A_{x,y}$ are the amplitudes (half-widths) of the pattern in the horizontal and vertical directions, $\omega _{x,y}$ are the angular frequencies of the pattern, and $\phi _{x,y}$ are the phase shifts which dictate the starting coordinates of the curve. By tuning these six parameters, any rectangular Lissajous curve can be generated, though this formulation restricts the pattern to be centered at the origin and orthonormal with the *x* and *y* basis vectors.

### Discrete recursive formulation

A simple version of the discrete linear recurrence relation describing 2D Lissajous curve waypoint generation was derived in^19^. This linear discrete recursive formula is a useful representation of the curve which is amenable to linear estimation and control frameworks.

Define the discrete state vector ${\textbf{x}}_{k}$ as follows:

9 $$\begin{aligned} {\textbf{x}}_{k} = \begin{bmatrix} x_{k}&{\dot{x}}_{k}&y_{k}&{\dot{y}}_{k} \end{bmatrix}^{\top }. \end{aligned}$$

This state is propagated according to the following linear model with sample period $\Delta t$:

10 $$\begin{aligned} {\textbf{x}}_{k} = {\textbf{A}}{\textbf{x}}_{k-1}, \end{aligned}$$

where

11 $$\begin{aligned} {\textbf{A}} = \begin{bmatrix} 1-\Big (\frac{\Delta t\omega _x}{2}\Big )^2 &{} \Delta t &{} 0 &{} 0 \\ -\Delta t {\omega _x}^2 &{} 1-\Big (\frac{\Delta t\omega _x}{2}\Big )^2 &{} 0 &{} 0 \\ 0 &{} 0 &{} 1-\Big (\frac{\Delta t\omega _y}{2}\Big )^2 &{} \Delta t \\ 0 &{} 0 &{} -\Delta t {\omega _y}^2 &{} 1-\Big (\frac{\Delta t\omega _y}{2}\Big )^2 \end{bmatrix}. \end{aligned}$$

The matrix ${\textbf{A}}$ is a second-order approximation of the state transition matrix which precisely discretizes the Lissajous curve. The desired amplitudes and phases of the pattern are used to derive the initial state conditions:

12 $$\begin{aligned} {\textbf{x}}_{0} = \begin{bmatrix} A_{x}\sin {(\phi _{x})} \\ \omega _{x}A_{x}\cos {(\phi _{x})} \\ A_{y}\sin {(\phi _{y})} \\ \omega _{y}A_{y}\cos {(\phi _{y})} \end{bmatrix}. \end{aligned}$$

### Features of Lissajous curves

**Figure 3:**
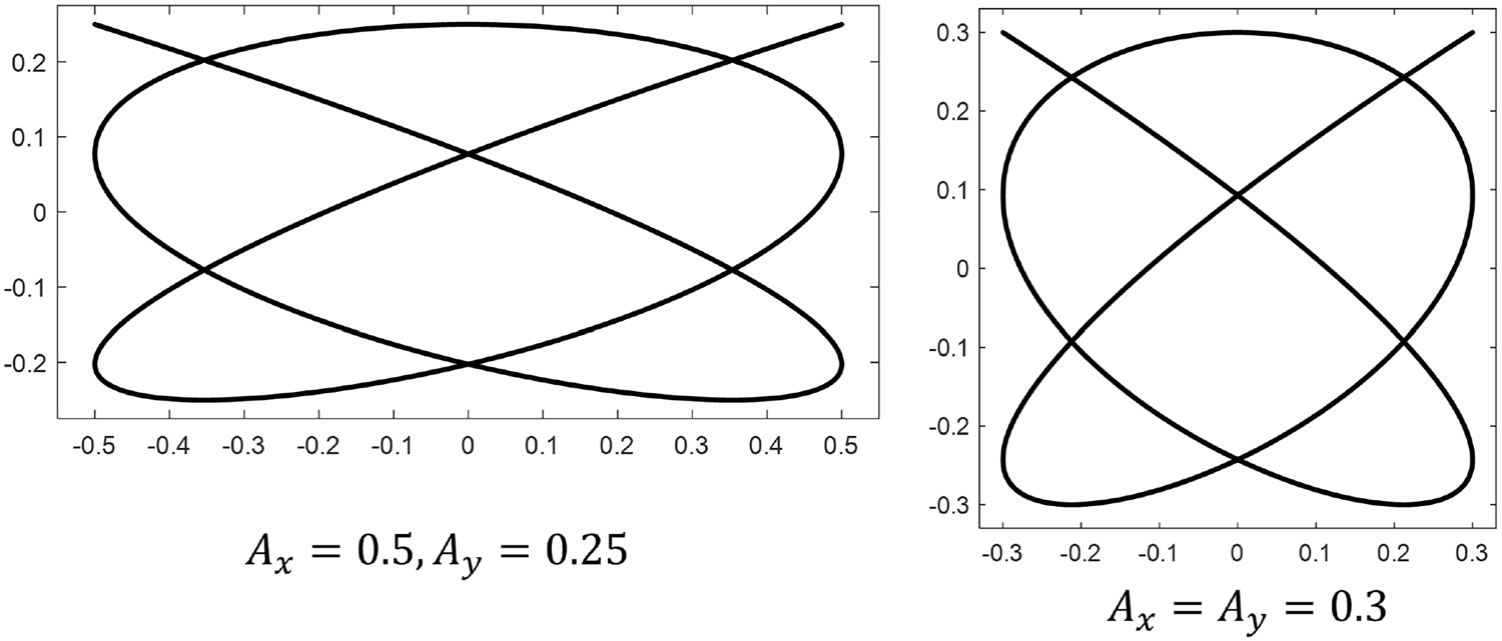
Effect of varying Lissajous amplitude. $\omega _x = 1$, $\omega _y = 0.8$, $\phi _x = 0$, $\phi _y = \pi /2$.

**Figure 4:**
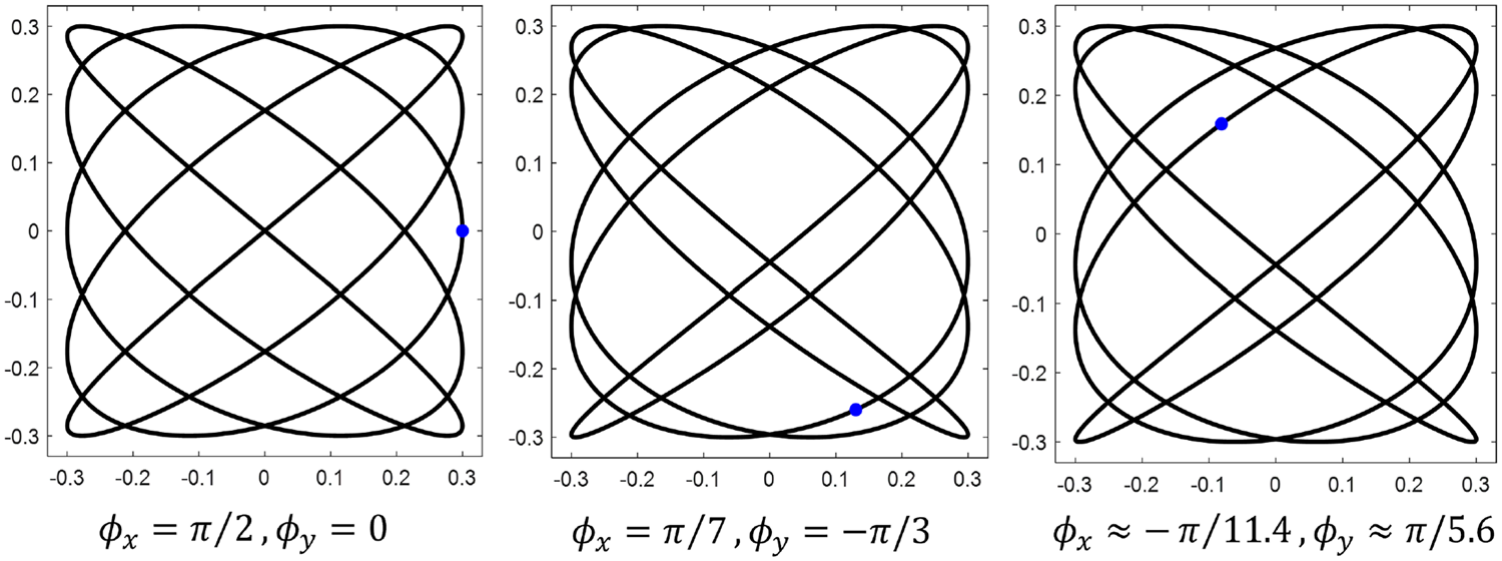
Effect of varying Lissajous phase shift. Blue dots denote figure starting point. $A_x = A_y = 0.3$, $\omega _x = 1$, $\omega _y = 0.8$.

**Figure 5:**
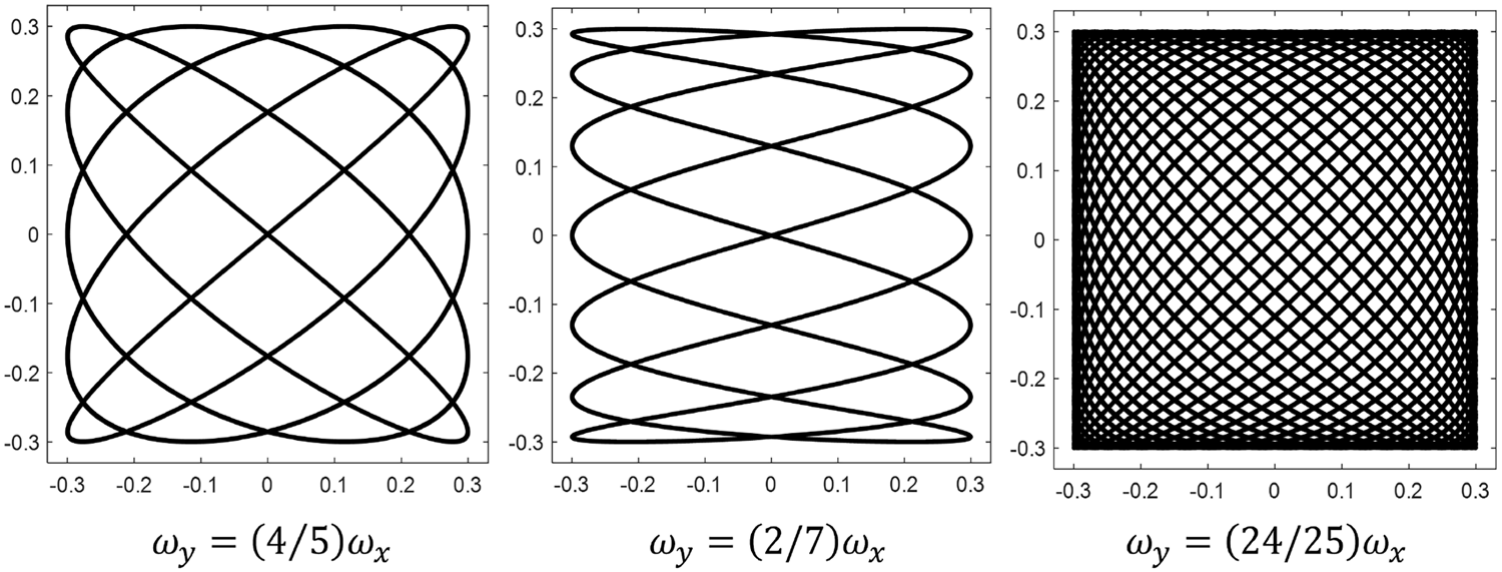
Effect of varying Lissajous frequency ratio. $A_x = A_y = 0.3$, $\phi _x = \pi /2$, $\phi _y = 0$.

**Figure 6:**
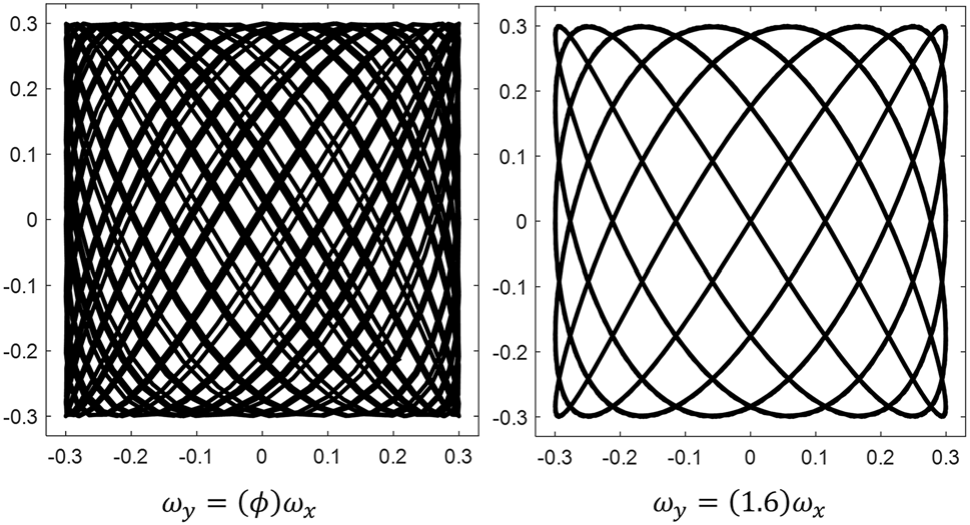
Irrational vs. rational frequency ratio. Note: in this figure, $\phi$ is the golden ratio, $(1+\sqrt{5})/2$. $A_x = A_y = 0.3$, $\phi _x = \pi /2$, $\phi _y = 0$.

In addition to the formulations above, a qualitative description of Lissajous curves is beneficial to better convey their utility for aerial search. Figures 3-6 are included here to visually demonstrate the effect of varying each of the key parameters in Eq. (8).

As Fig. 3 demonstrates, the amplitude parameters $A_x$ and $A_y$ of Eq. (8) can be adjusted to match the width and length of a rectangular search space.

While the effect of the amplitude parameters on the shape of the curve is relatively straight-forward, the effect of the phase shifts $\phi _x$ and $\phi _y$ is more complex. These parameters determine the start point of the pattern and they also have an influence on the size of gaps in the curve. This is demonstrated by Fig. 4.

When the area of the smallest gap collapses to zero, the pattern takes on a lower order of complexity (this can be seen in Fig. 3, where $\phi _x$ approaches zero and $\phi _y$ approaches $\pi /2$). Furthermore, as Fig. 4 shows, a given combination of $\phi _x$ and $\phi _y$ does not uniquely specify a Lissajous curve. For a fixed set of amplitudes and angular frequencies, there are infinitely many $\phi _{x,y}$ combinations that yield the same pattern, but there are infinitely more phase shifts which produce different curves. However, the number of intersections in a Lissajous curve (i.e. its level of complexity) can only be controlled by varying the frequency parameters $\omega _x$ and $\omega _y$. More specifically, it is the ratio of the angular frequencies in the x and y directions that governs complexity, as the absolute value of these parameters affects only the “speed” of the pattern. Figure 5 demonstrates examples of three different frequency ratios.

It is worth noting that frequency ratios near one cover a space with higher rotational symmetry than smaller ratios: this is evident when comparing the 2/7 pattern with the 24/25 pattern. Finally, it can be shown without much difficulty that irrational frequency ratios result in curves which never repeat. Figure 6 contrasts an irrational frequency ratio with a nearly equal rational one.

## Analysis of LPs for aerial search

### Formula generalization

The first original contribution of this work is a generalization of the LP which allows the curve to be rotated and shifted in 2D space.

#### Continuous

The generalized continuous formula for a Lissajous curve is given below:

13 $$\begin{aligned} \begin{bmatrix} X(t) \\ Y(t) \end{bmatrix} = \begin{bmatrix} \cos {\theta } &{} -\sin {\theta } \\ \sin {\theta } &{} \cos {\theta } \end{bmatrix} \begin{bmatrix} A_{x}\sin {(\omega _{x}t+\phi _{x})} \\ A_{y}\sin {(\omega _{y}t+\phi _{y})} \end{bmatrix} + \begin{bmatrix} {\overline{X}} \\ {\overline{Y}} \end{bmatrix}, \end{aligned}$$

where ${\overline{X}}$ and ${\overline{Y}}$ are the offsets of the pattern in the *x* and *y* directions, respectively, and $\theta$ is the angle by which the bounding rectangle of the pattern is oriented. This formulation offers more versatility in constructing Lissajous curves than Eq. (8).

#### Discrete

Rotating and translating a Lissajous curve using the discrete recursive representation is more challenging than in the continuous case. While a more thorough derivation can be found in “Appendix A”, the result is given here. Defining the state vector as in Eq. (9), the state is propagated by the following discrete linear model:

14 $$\begin{aligned} {\textbf{x}}_{k} = \mathbf {\Phi }{\textbf{x}}_{k-1} + \mathbf {\Psi }, \end{aligned}$$

where $\mathbf {\Phi }=\mathbf {\mathcal {R}}{\textbf{A}}\mathbf {\mathcal {R}}^{\top }$, $\mathbf {\Psi } = ({\textbf{I}}-\mathbf {\Phi })\overline{{\textbf{x}}}$, $\overline{{\textbf{x}}} = [{\overline{X}} \quad 0 \quad {\overline{Y}} \quad 0]^\top$, ${\textbf{A}}$ is defined in Eq. (11), and

15 $$\begin{aligned} \mathbf {\mathcal {R}} = \begin{bmatrix} \cos {\theta } &{} 0 &{} -\sin {\theta } &{} 0 \\ 0 &{} 1 &{} 0 &{} 0 \\ \sin {\theta } &{} 0 &{} \cos {\theta } &{} 0 \\ 0 &{} 0 &{} 0 &{} 1 \end{bmatrix}. \end{aligned}$$

This also requires that the initial state be transformed according to:

16 $$\begin{aligned} {\textbf{x}}_{0} = \mathbf {\mathcal {R}} \begin{bmatrix} A_{x}\sin {(\phi _{x})} \\ \omega _{x}A_{x}\cos {(\phi _{x})} \\ A_{y}\sin {(\phi _{y})} \\ \omega _{y}A_{y}\cos {(\phi _{y})} \end{bmatrix} + \overline{{\textbf{x}}}. \end{aligned}$$

This construction of Lissajous curves allows any pattern to be generated at any location and orientation in the plane.

### Expected distance to a uniform random target

Designing an “optimal” Lissajous search pattern is an open-ended task. One must take into account many factors, including the assumed prior belief about the target, the dimensions of the search space, the maximum duration of the search, and so on. To begin to approach this problem analytically, several assumptions must be made about the circumstances of the search problem at hand. The developments of this section make the following assumptions about the search scenario:

1. No prior knowledge is available about the target’s location, except that...
2. ...its distribution has 90^∘^ rotational symmetry, and...
3. ...the target must be located within the confines of a rectangular search space...
4. ...whose dimensions are one unit by one unit, and...
5. ...the searcher starts from rest in the southeast corner.

For generality, all variables in the following developments are dimensionless. To further simplify, let $\theta = 0$ and ${\overline{X}} = {\overline{Y}} = 0$ without loss of generality.

Assumption 3 is inherently compatible with a Lissajous curve, as these patterns are always inscribed by a rectangle. From assumption 4, the values of $A_x$ and $A_y$ in Eq. (13) must be 0.5. In order to enforce assumption 5, ${\textbf{x}}_{0}$ must be $[0.5 \quad 0 \quad -0.5 \quad 0]^\top$. From this, it follows that $\phi _{x} \equiv \frac{\pi }{2}(4n-3)$ and $\phi _{y} \equiv -\frac{\pi }{2}(4n-3)$, or for simplicity, $\phi _{x} \equiv \frac{\pi }{2}$ and $\phi _{y} \equiv -\frac{\pi }{2}$. Finally, to simplify the problem formulation in keeping with assumption 2, let the frequency ratio $r_\omega \in {\mathbb {R}}_{+}^{*}$ be introduced and defined as follows:

17 $$\begin{aligned} r_\omega = \frac{\omega _x}{\omega _y}. \end{aligned}$$

This is the single most critical parameter in varying the complexity of an LP, as described in section “Features of Lissajous curves”. The assumption that the target distribution has 90^∘^ rotational symmetry allows the range of values taken by $r_\omega$ to be truncated to the half-open interval (0, 1]. Thus, reflecting assumptions 2–5, the continuous Lissajous formulation of Eq. (13) can be reduced to the following:

18 $$\begin{aligned} \begin{bmatrix} X_S(t) \\ Y_S(t) \end{bmatrix} = \begin{bmatrix} 0.5\sin {(r_\omega \omega _{y}t+\frac{\pi }{2})} \\ 0.5\sin {(\omega _{y}t-\frac{\pi }{2})} \end{bmatrix} = \begin{bmatrix} 0.5\cos {(r_\omega \omega _{y}t)} \\ -0.5\cos {(\omega _{y}t)} \end{bmatrix}. \end{aligned}$$

(Note: the *S* subscripts on *X* and *Y* denote the *x* and *y* positions of the *searcher*.) Therefore, with only a few reasonable and nearly trivial assumptions, a nine-dimensional parametric optimization space is reduced to only two.

While assumptions 2–5 affect the parameters of the LP used in a search scenario, the first assumption relates to the assumed target distribution. If no information is available apart from the boundaries of the search domain, the distribution chosen to probabilistically represent the location of a target must maximize the differential entropy of the target. It can be shown^23^, and is also fairly intuitive, that the uniform distribution accomplishes this. If the support of the target location is known, the boundaries of the search pattern should be made to match in order to maximize the efficiency of the search. Since assumption 4 restricts the dimensions of the Lissajous curve, the probability density function (PDF) of the target’s location $X_T$, $Y_T$ must be:

19 $$\begin{aligned} f_{X_T,Y_T}(x_T,y_T) = {\left\{ \begin{array}{ll} 1, \quad -0.5<(x_T,y_T)<0.5 \\ 0, \quad else \end{array}\right. }. \end{aligned}$$

Analytically optimizing an LP subject to the above assumptions and constraints becomes a matter of determining which value(s) of $r_\omega$ result in the fastest completion of a search mission. Without inserting any additional information about the modality of target detection, it may be reasonably assumed that such an optimal pattern should be made to minimize the expected distance between the searcher and the target, averaged over all time. The distance between the searcher and the target in two dimensions is simply

20 $$\begin{aligned} d(x_S,y_S,x_T,y_T) = \sqrt{(x_S-x_T)^2+(y_S-y_T)^2} \end{aligned}$$

(altitude is removed from the problem since it is assumed that the searcher can only identify the target when it is nearly directly above it). The expected value of this distance is:

21 $$\begin{aligned} \begin{aligned} E[d(x_S,y_S)]&= \int _{\mathcal {D}} f_{X_T,Y_T}(x_T,y_T)d(x_S,y_S,x_T,y_T) dA \\ {}&= \int _{-0.5}^{0.5}\int _{-0.5}^{0.5} \sqrt{(x_S-x_T)^2+(y_S-y_T)^2} dx_Tdy_T \end{aligned}. \end{aligned}$$

**Figure 7:**
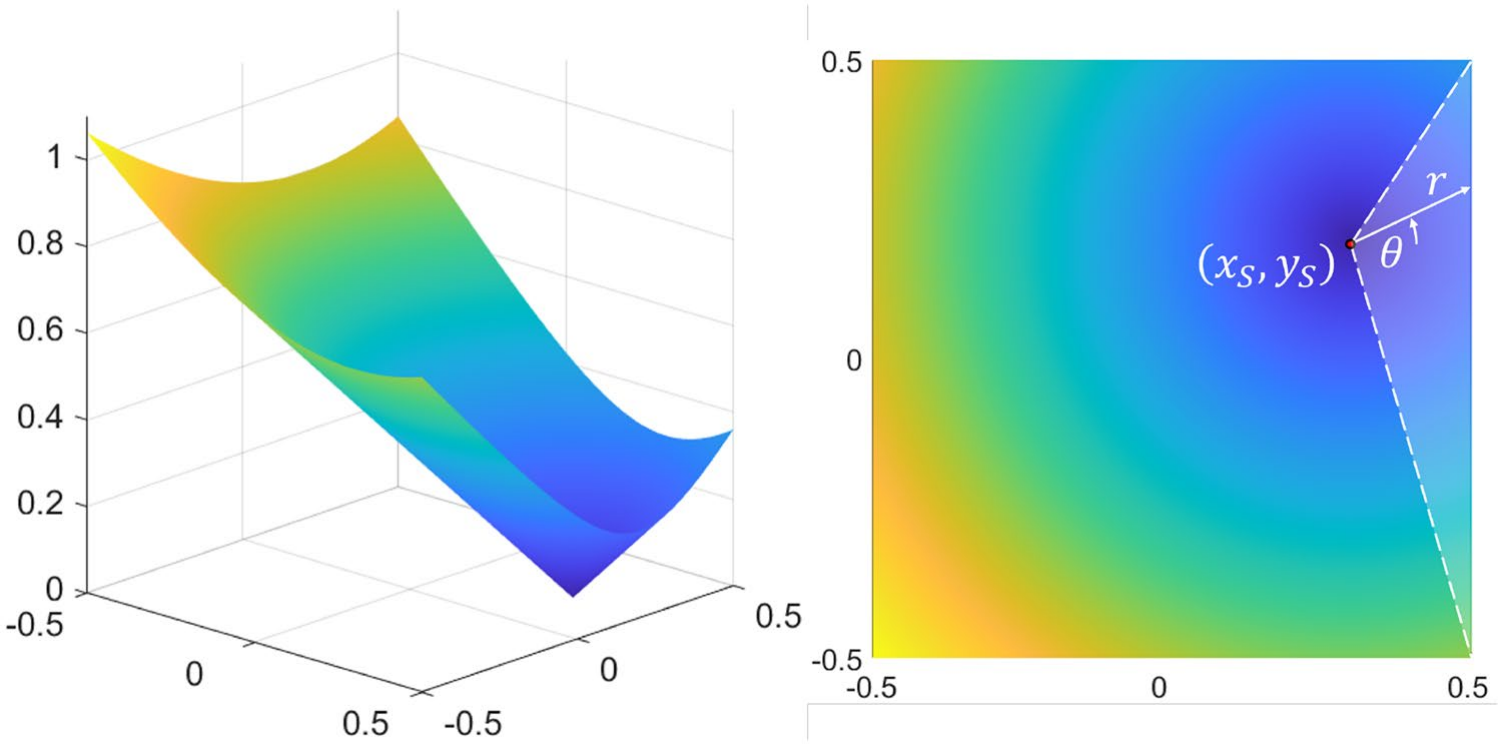
Surface of integration for distance function $d(x_S,y_S,x_T,y_T)$.

This is a notoriously challenging integral. However, it may be noted that the integrand is more easily represented in polar coordinates, in which case the integral becomes:

22 $$\begin{aligned} \int _{\mathcal {D}}\int r dA = \int _{\mathcal {D}}\int (r) rdrd\theta = \int _{\mathcal {D}}\int r^2 dr d\theta \end{aligned}$$

where $r=\sqrt{(x_S-x_T)^2+(y_S-y_T)^2}$. While the operation is now simpler, the region of integration is no longer constant. It is useful to visualize the surface being integrated to gain insight about the integration limits; this visualization is provided in Fig. 7.

It can be shown that the integral of the white-highlighted triangular region of the figure is:

23 $$\begin{aligned} \int _{-\tan ^{-1}{\big (\frac{1/2+y_S}{1/2-x_S}\big )}}^{\tan ^{-1}{\big (\frac{1/2-y_S}{1/2-x_S}\big )}} \int _{0}^{\big (\frac{1/2-x_S}{\cos {\theta }}\big )} r^2 dr d\theta . \end{aligned}$$

**Figure 8:**
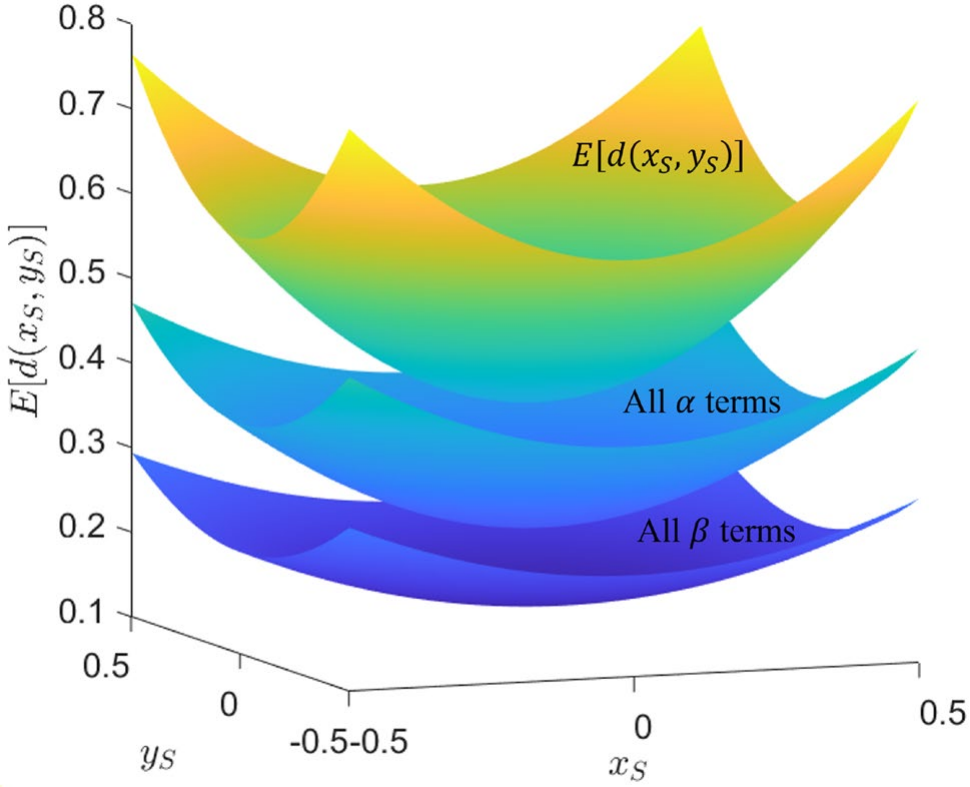
Expected distance between searcher and randomly located target.

This is worked out in “Appendix B”. Adding the result of this integration with those of the remaining three regions gives the following result for the expected distance $E[d(x_S,y_S)]$ between a uniformly randomly distributed target location on the unit square and a given searcher location $(x_S,y_S)$:

24 $$\begin{aligned} \begin{aligned} E[d(x_S,y_S)]&= \frac{1}{3}\Big [ \alpha (x_S,y_S) + \alpha (x_S,-y_S) + \alpha (-x_S,y_S) + \alpha (-x_S,-y_S) \\&+ \beta (-x_S,-y_S) + \beta (-x_S,y_S) + \beta (-y_S,x_S) + \beta (-y_S,-x_S) \\&+ \beta (x_S,y_S) + \beta (x_S,-y_S) + \beta (y_S,-x_S) + \beta (y_S,x_S)\Big ], \end{aligned} \end{aligned}$$

where $\alpha (a,b) = \big (\frac{1}{2}+a\big )\big (\frac{1}{2}+b\big )\sqrt{\big (\frac{1}{2}+a\big )^2 + \big (\frac{1}{2}+b\big )^2}$ and $\beta (a,b) = \big (\frac{1}{2}+a\big )^3\tanh ^{-1}{\Bigg (\frac{\sqrt{\big (1/2+a\big )^2 + \big (1/2+b\big )^2} - \big (1/2+a\big )}{\big (1/2+b\big )}\Bigg )}$. This expected distance function is minimized to a value of 0.3826 when $x=y=0$, maximized to a value of 0.7639 when $x=y=\frac{1}{2}$, and is rotationally symmetric. The surface described by Eq. (24) is plotted in Fig. 8. This figure also depicts the surface visually decomposed into the $\alpha$ and $\beta$ terms alone (It may be helpful to note that $\alpha$ and $\beta$ are not significant functions themselves but are just defined here for compactness. They are plotted in the figure to show the relative contribution of each to the total expected distance.).

Let ${\overline{D}}(r_\omega )$ represent the *average expected distance* from a searcher to a random target over a long period of time:

25 $$\begin{aligned} {\overline{D}}(r_\omega ) = \lim _{T \rightarrow \infty } \frac{1}{T}\int _0^T E[d(x_S(r_\omega ,t),y_S(r_\omega ,t))] dt. \end{aligned}$$

**Figure 9:**
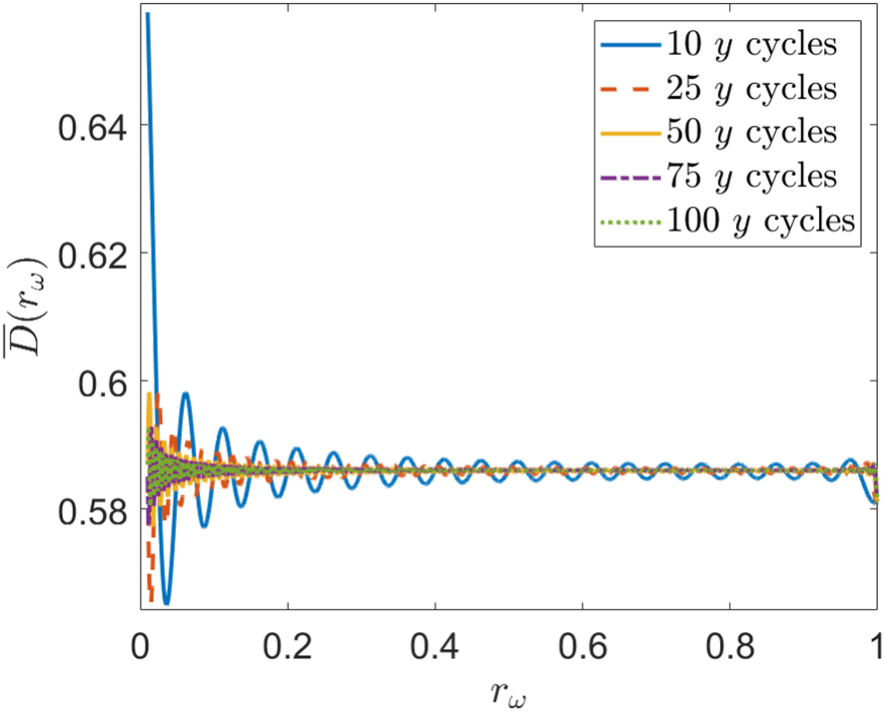
Average expected distance vs. frequency ratio for several search cycles.

If a value of $r_\omega$ can be found which minimizes ${\overline{D}}(r_\omega )$, it may follow that an efficient Lissajous search pattern should be designed with that minimizing frequency ratio, as it guarantees that the searcher will be as near to the target (on average) as possible. However, combining Eqs. (13) and (24) is extremely unwieldy and the integral of Eq. (25) is intractable. Nevertheless, it is possible to evaluate ${\overline{D}}(r_\omega )$ numerically. The average expected distance was computed over 10,000 irrational values of $r_\omega$ from 0 to 1 to ensure that the pattern never repeated. This was conducted for five values of *T* ranging from $10(\frac{2\pi }{\omega _y})$ to $100(\frac{2\pi }{\omega _y})$ as shown in Fig. 9 and summarized in Table 1. Though it cannot be proven analytically, it is clear from the figure that ${\overline{D}}(r_\omega )$ converges to a value of approximately 0.586 as $T \rightarrow \infty$. This is further supported by Table 1. Therefore, we conjecture that over a long time, the average distance between a Lissajous searcher and any target uniformly randomly located within a square region is the same regardless of the shape of the LP. This statement is further supported by the fact that the average expected *squared* distance can be proven to be a constant $\frac{5}{12}$ on the support of the unit square (see “Appendix C”).

**Table 1:** Mean and standard deviation of ${\overline{D}}(r_\omega )$ Over $r_\omega$ for varying search pattern lengths.

	Number of *y* cycles				
	10	25	50	75	100
Mean	0.5861	0.5858	0.5860	0.5860	0.5860
S.D.	0.0061	0.0021	0.0011	0.00075	0.00057

While it is an interesting and somewhat unintuitive result that the average expected distance to a randomly located target is independent of the searcher’s Lissajous path, it does not aid in the design of an optimal Lissajous search pattern. Furthermore, this approach cannot account for the technicalities of target detection (i.e. at what distance between a target and a searcher can the target be considered found?). For these reasons and others, practical Lissajous search pattern design must be done numerically. Before this is detailed in section “Numerical assessment and design of Lissajous search patterns”, it is critical to know the relationship between an LP’s parameters and its average speed in order to conduct fair and consistent numerical simulations. This can be thought of as a normalization of Lissajous patterns. For example, if numerical optimization shows that low values of $r_\omega$ appear to perform the best, yet those frequency ratios cause a searcher to travel much faster, on average, than other values of $r_\omega$, the comparison would be misleading. Therefore, a derivation of the average speed of a Lissajous searcher is first appropriate.

### Average speed of a Lissajous searcher

The instantaneous speed (i.e. the magnitude of the velocity vector) of a continuously formulated LP subject to the assumptions of section “Expected distance to a uniform random target” is given as follows:

26 $$\begin{aligned} \begin{aligned} s(t)&= \sqrt{{\dot{x}}^2(t)+{\dot{y}}^2(t)} \\&=\sqrt{\Big (0.5\omega _x\sin {(\omega _xt)}\Big )^2+\Big (-0.5\omega _y\sin {(\omega _yt)}\Big )^2}, \end{aligned} \end{aligned}$$

where dots denote time derivatives. The average speed over some period *T* is then

27 $$\begin{aligned} {\overline{s}} =\frac{\omega _y}{2T}\int _0^T \sqrt{r_\omega ^2\sin ^2{(r_\omega \omega _yt)}+\sin ^2{(\omega _yt)}} dt. \end{aligned}$$

This integral is again intractable. However, approximations can be considered to varying degrees of accuracy. The alpha-max-plus-beta-min algorithm commonly used to speed up magnitude computations in digital signal processing^24–26^ offers the following approximation for *s*(*t*):

28 $$\begin{aligned} s(t) \approx \alpha |\max {\{{\dot{x}}(t),{\dot{y}}(t)\}}|+\beta |\min {\{{\dot{x}}(t),{\dot{y}}(t)\}}|, \end{aligned}$$

where optimal values of $\alpha = 0.96$ and $\beta = 0.40$ guarantee an error of less than 4%. However, this algorithm requires that the larger of ${\dot{x}}(t)$ and ${\dot{y}}(t)$ be known for all *t*—knowledge which cannot be guaranteed for a general Lissajous curve. Thus, a symmetric approximation is sought. “Appendix D” shows that the optimal symmetric linear approximation of the Pythagoream theorem is:

29 $$\begin{aligned} s(t) \approx 0.779\big (|{\dot{x}}(t)|+|{\dot{y}}(t)|\big ), \end{aligned}$$

with error no greater than $22.1\%$. While it introduces a larger error margin, this approximation enables a much simpler estimate of the average speed of a Lissajous searcher.

The estimated average speed ${\overline{s}}$ is determined by

30 $$\begin{aligned} {\overline{s}} \approx \frac{0.779}{2T}\int _0^T \big (|\omega _x\sin {(\omega _xt)}| + |\omega _y\sin {(\omega _yt)}|\big ) dt. \end{aligned}$$

Let the interval *T* over which the speed is being averaged allow for a complete number of periods in both the *x* and *y* directions:

31 $$\begin{aligned} T = \frac{2\pi m}{\omega _x} = \frac{2\pi n}{\omega _y}, \end{aligned}$$

where $m \in {\mathbb {Z}}$ is the number of cycles in the *x* and $n \in {\mathbb {Z}}$ is the number of cycles in the *y* (While these developments are technically only valid for rational frequency ratios $r_\omega = \frac{m}{n}$, the errors induced when applying the result to irrational frequency ratios are negligible.). It can be shown without much difficulty that

32 $$\begin{aligned} \int _0^{\frac{2\pi p}{\omega }} |\sin {(\omega t)}| dt = 2p\int _0^{\frac{\pi }{\omega }} \sin {(\omega t)} dt = \frac{4p}{\omega }, p = 1,2,\ldots \end{aligned}$$

and so,

33 $$\begin{aligned} {\overline{s}} \approx \frac{0.779}{4\pi n}\omega _y(4n + 4m) = \frac{0.779}{\pi }\omega _y(1 + r_\omega ). \end{aligned}$$

Solving for $\omega _y$ (with $\omega _x$ following from Eq. (17)) gives (More generally, for an LP with arbitrary amplitudes, ${\overline{s}} \approx \frac{1}{\pi }\Big [-1 + \sqrt{2} - \sinh ^{-1}{(1)} + \sqrt{2}\tanh ^{-1}{\Big (\frac{1}{\sqrt{2}}\Big )}\Big ]\Big (A_x \omega _x + A_y \omega _y \Big )$.)

34a $$\begin{aligned} \omega _y&\approx \frac{\pi {\overline{s}}}{0.779(1 + r_\omega )}, \end{aligned}$$

34b $$\begin{aligned} \omega _x&= r_\omega \omega _y. \end{aligned}$$

**Figure 10:**
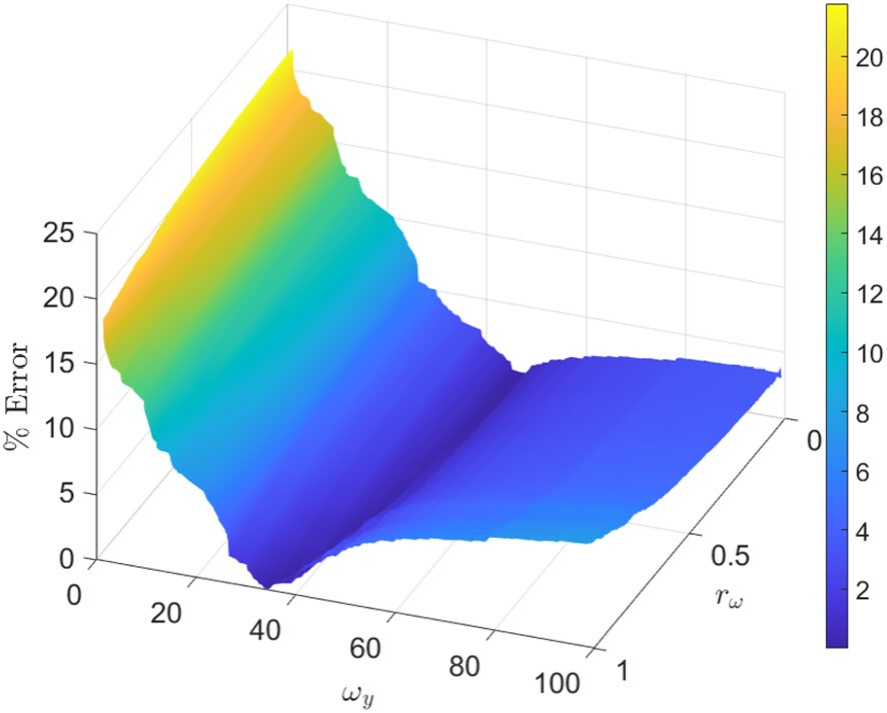
Percent error in estimated speed using Eq. (33) vs. numerical ground truth.

Therefore, for a desired average Lissajous searcher speed of ${\overline{s}}$ and a frequency ratio of $r_\omega$, it can be guaranteed that choosing $\omega _y$ and $\omega _x$ using Eqs. (34) will result in a true average searcher speed with an error of no more than $\approx 22\%$. To confirm this, 40,000 LPs were generated with random values of $\omega _y$ ranging from 1 to 100 and frequency ratios ranging from 0 to 1. Equation (33) was applied to solve for the estimated path speed and this was compared to the “true” (numerical) measure. The percent-error surface is plotted in Fig. 10. Note that the upper limit of the error indeed approaches the theoretical limit as $\omega _y \rightarrow 0$.

## Numerical Assessment and design of Lissajous search patterns

### Monte Carlo simulation

Given the analytical complexity of designing LPs, a Monte Carlo simulation technique was implemented to numerically achieve Lissajous flight path optimization. As described above, the goal of LP design is to choose a frequency ratio $r_\omega$ that produces the most effective search path subject to the assumptions and constraints of section “Expected distance to a uniform random target”. However, with a numerical/simulation approach, one can bypass the task’s analytical intractability to deliver waypoints for aerial vehicles conducting real-world search missions.

Multiple aerial searching scenarios can be simulated simultaneously by populating a unit square space with uniformly randomly generated targets, represented by their 2D Cartesian coordinates. Previous work^22^ showed that 10,000 random targets are generally sufficient to achieve convergence of outcomes for the constraints of the scenarios addressed here. The Lissajous search path is then numerically represented by a sequence of dense waypoints generated by Eqs. (13) or (14). To ensure equitable search pattern comparison, waypoints were generated at resolutions that delivered constant average Lissajous speeds by calculating the appropriate values of $\omega _x$ and $\omega _y$ for each pattern. Although an approximate analytical approach for this was derived in section “Average speed of a Lissajous searcher”, a pre-processed numerical lookup table was instead used here to reduce potential maximum error from near 22% to only fractions of a percent. At each waypoint, the detection state of each target was evaluated. Unlike in the analytical case, it is possible in simulation to account for the modality of target detection when assessing the performance of a search pattern. The simulated detector checks which targets lie in a rectangular field of view (FOV) at each waypoint in order to emulate the detection of a small target by an optical sensor. To limit the number of additional parameters introduced in the optimization problem, we assume a square FOV as projected onto the ground plane with side length $l_f$. Detection is then accomplished efficiently in a two-step process. First, the Euclidean distance between the searcher and all targets is computed and targets whose distance from the searcher exceeds $\sqrt{2}l_f$ (the radius of the circle which circumscribes the FOV) are removed from further consideration. The much smaller subset of targets which lie near the agent at a given waypoint are determined to be in the FOV by first transforming their coordinates into the searcher’s reference frame. If the magnitude of any of these transformed coordinates is less than $l_f/2$, the corresponding target is considered to have been detected.

**Figure 11:**
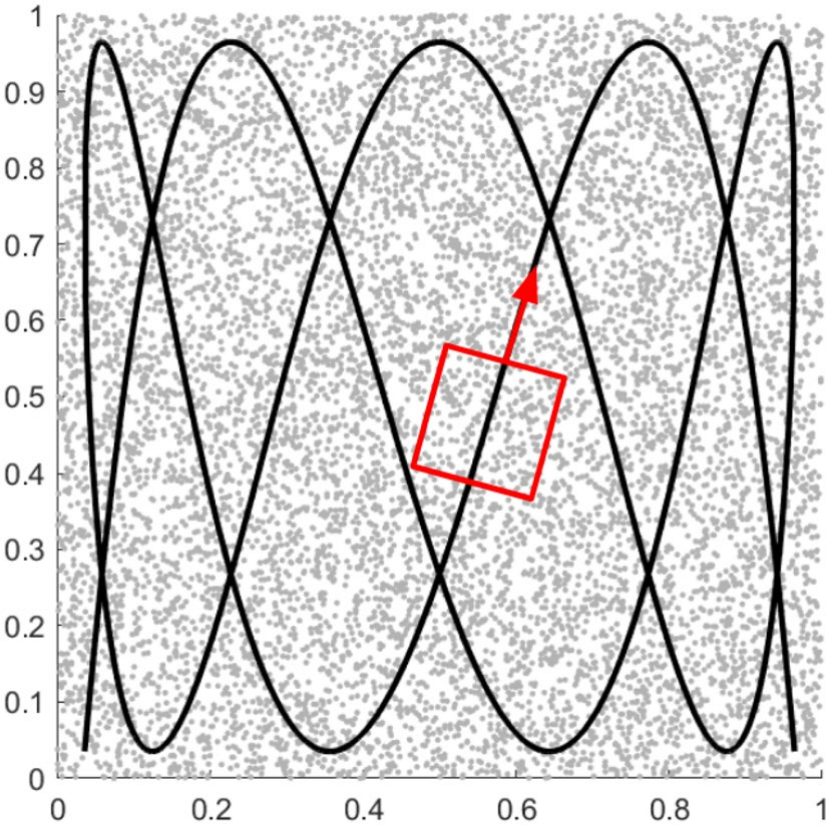
Simulated UAS with square FOV navigating the search space along a $r_\omega = 0.3$ Lissajous path.

Figure 11 illustrates such a search scenario with uniform random target locations, searcher path, and FOV displayed. As one might expect, there is an important interplay between the maximum gap size in an LP and the size of the FOV. This relationship, investigated more thoroughly in the results of^22^, is taken into account in the statistical modeling of section “Predictive modeling for Lissajous search pattern design”.

**Figure 12:**
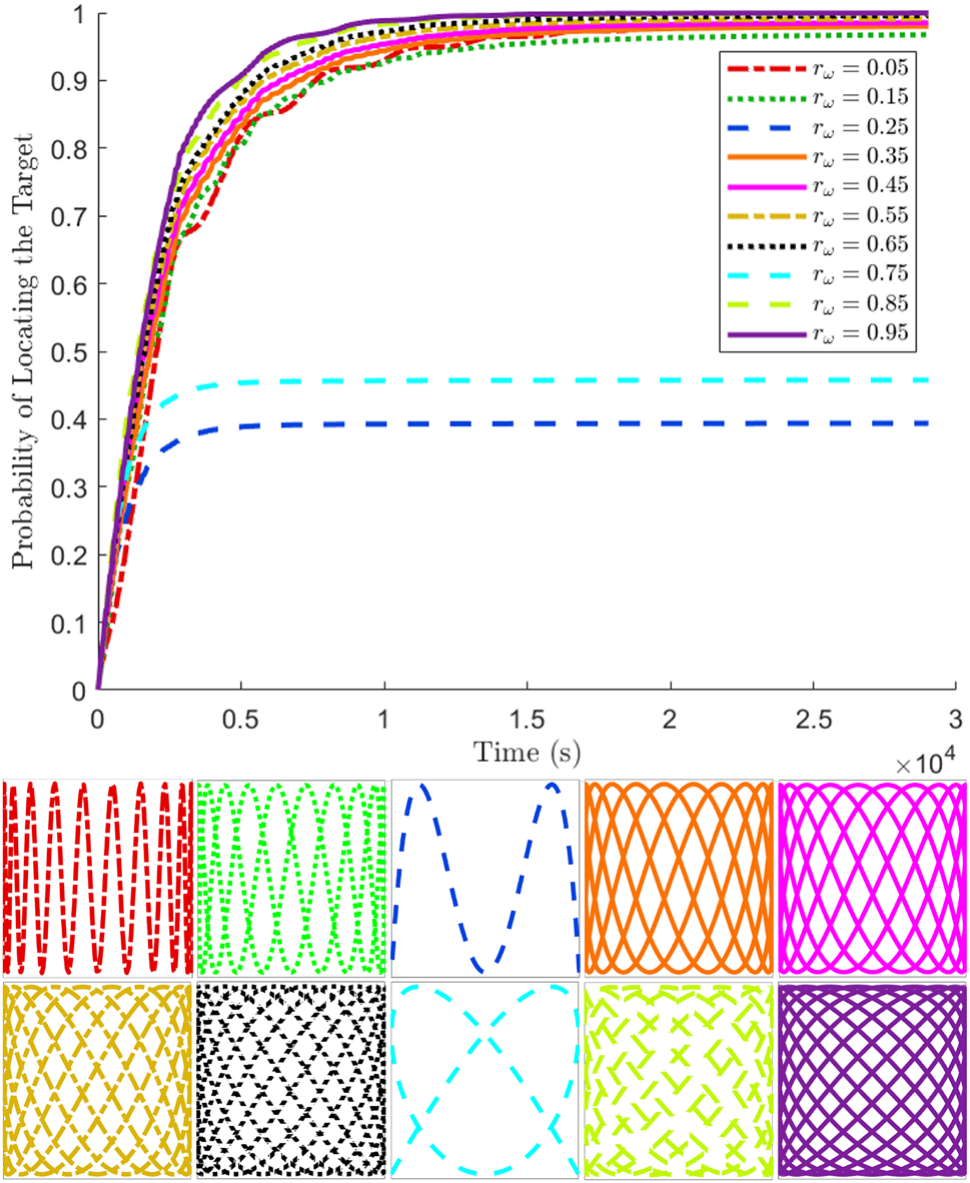
Monte Carlo simulation results showing examples of search CDFs and their corresponding Lissajous curves over a range frequency ratios. Curves should be read in increasing order from upper-left to lower-right. For these simulations, $\tau = 20$.

Even when a target lies in a UAS’s FOV, no real-world detector can deliver 100% certainty of finding it, as sensor-based detection is always ultimately stochastic. For this reason, a probabilistic model for detection was chosen based on^27^ to transform the time $t_d$ a target spends in the detectable region (i.e. FOV) of a searcher into a probability of having been detected. The longer a particular target is inside the FOV, the more likely it is to be seen by the simulated UAS. This probability is modeled as

35 $$\begin{aligned} p(t_d) = 1-\exp {\Big (-\frac{t_d}{\tau }\Big )}, \end{aligned}$$

where $\tau$ is a scaling time constant which holistically models the sensitivity of the detector (a smaller time constant corresponds to a more robust detector). At the conclusion of a simulation, each target has an associated probability of having been detected by the end of a search. Additional insight can be obtained by generating the cumulative density function (CDF) $F_T(t)$ which describes the probability that the time at which a uniformly randomly located target is found (detected) at a time *T* is less than *t*. Examples of Lissajous search pattern CDFs are shown in Fig. 12. While this figure is intended to serve primarily as a qualitative demonstration of how the performance of search patterns affects the shape of their CDFs, an accompanying quantification of this figure is provided in Table 2, which gives the CDFs’ means and final values.

**Table 2:** Mean and final value of CDFs for example Lissajous patterns.

$r_\omega$	0.05	0.15	0.25	0.35	0.45	0.55	0.65	0.75	0.85	0.95
CDF mean value	0.877	0.873	0.380	0.888	0.898	0.908	0.914	0.443	0.926	0.928
CDF final value	0.984	0.968	0.394	0.980	0.986	0.991	0.996	0.458	0.999	1.000

The CDF is a helpful visual representation of a particular LP’s performance in the time domain, but it does not provide a single summative value to use as an objective function in the optimization problem. The mean or final value of the CDF may be reasonable evaluation metric candidates, but neither of these parameters captures much information about the early behavior of the pattern. For this reason, an evaluation criterion which reflects the insightful shape of the CDF is desired for comparison between LPs.

### Evaluation criteria

Previous work^21^ proposed the use of the area under the CDF (AUC) as a qualitative summary metric for characterizing the effectiveness of a search pattern. If a pattern is particularly robust, its CDF will demonstrate a steep rise early on, contributing to a large overall AUC. However, the challenge introduced by this metric is the arbitrariness of the upper limit of integration. It was formerly proposed that this limit should be chosen as the time at which a certain acceptable threshold for the probability of detecting a target has been achieved. Maximizing the AUC has practical utility in providing an optimization objective, but it lacks a clear underlying mathematical interpretation relating to the stochastic problem at hand. In this work, we instead propose to use the area above the CDF (AAC) as an objective function. Maximizing the AUC and minimizing the AAC achieve the same goal, but using the AAC has two distinct advantages over using the AUC: (1) the arbitrariness of a cutoff for integration is eliminated since a CDF always converges to one (This is always true if there are no gaps when the FOV is swept out over the search pattern.), and (2) the area above a CDF which is defined strictly over a non-negative domain is in fact equal to the mean of the distribution. This gives meaning and mathematical significance to the AAC as a criterion for evaluating a Lissajous search pattern.

**Figure 13:**
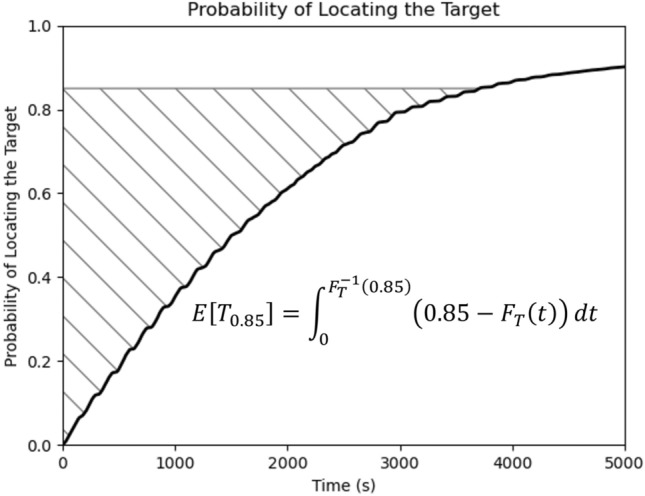
A CDF evaluation where the mission’s desired certainty threshold is 85% rather than 100%.

The area above the CDF, or the mean of the distribution, can be shown^28^ to be:

36 $$\begin{aligned} E[T] = \int _{0}^{\infty }(1-F_T(t)) \, \, dt, \end{aligned}$$

where *E*[*T*] is the time at which a uniformly randomly located target can be expected to be found by a UAS searcher flying on a prescribed Lissajous path. Therefore, smaller *E*[*T*] values are associated with more effective frequency ratios for pattern design. It is noteworthy that the upper integral bound in Eq. (36) need not necessarily be $\infty$. If a mission highly prioritizes the speed with which a target is located over the certainty of finding the target at all, a lower certainty threshold may be acceptable in which case the upper bound of the integral would change. If the desired certainty level for mission success is $\alpha$, Eq. (36) becomes

37 $$\begin{aligned} E[T_\alpha ] = \int _{0}^{F_T^{-1}(\alpha )}(\alpha -F_T(t)) \, \, dt, \end{aligned}$$

where now $E[T_\alpha ]$ can be described as “the expected time at which a search mission for a uniformly randomly located target is successful”. Figure 13 demonstrates this concept for $\alpha = 0.85$, a typical certainty threshold for urgent search missions. Previous work has shown that Lissajous curves are particularly advantageous over traditional deterministic patterns in such scenarios when 100% certainty of locating the target is not required^22^.

### Key findings

**Figure 14:**
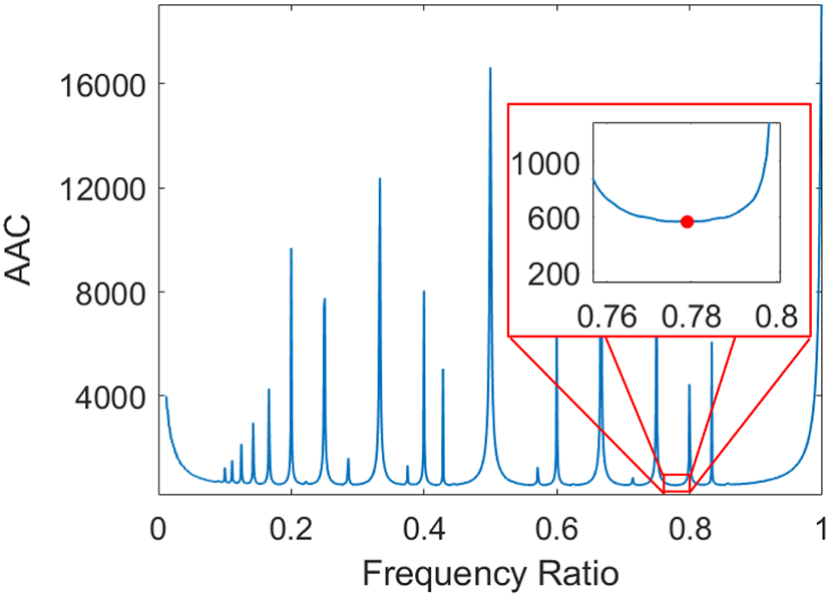
Frequency ratio decision plot showing the expected time to complete a search mission for every simulated LP (1000 frequency ratios tested). Optimal $r_\omega$ shown at $\sim 0.779$ with $E[T_\alpha ] = 564.926$.

Monte Carlo simulations were conducted as described above. Given the assumed $90^{\circ }$ rotational symmetry of the assumed target distribution, only frequency ratios spanning the interval (0, 1] require investigation, thus increasing the resolution of numerical optimization without introducing unnecessary computational burden. Figure 14 shows the dependency of LP performance on frequency ratio for $\alpha = 0.85$.

The absolute minimum $E[T_\alpha ]$ over this range naturally corresponds to the most effective frequency ratio for an LP, where any local minimum is a well-designed pattern and any local maximum is poorly suited for aerial search. One key finding from this approach is the recurring poor performance of Lissajous paths with near-rational frequency ratios. The large spikes at rational intervals imply that a higher degree of irrationality in frequency ratio is indicative of higher-performing LPs. This is an intuitive result in light of the fact that irrational patterns will never repeat, so “more rational” patterns will wastefully overlap themselves more often. Furthermore, rational frequencies are prone to leaving more unsearched areas in the search space as shown in Fig. 6, thus resulting in higher mission completion times. With these results, the task of designing a Lissajous search path becomes simply selecting the frequency ratio corresponding to the optimal minimum $E[T_\alpha ]$ value. This pattern will be the most effective flight path given the parameters of the search scenario that was simulated.

While the simulation method yields important design results, it is not without drawbacks. The numerical optimization process is computationally intensive, discouraging real-world implementation where deployment speed is of high priority. For this reason, a predictive modeling technique was considered for frequency ratio selection which bears the bulk of the computational cost up-front at model fitting, but almost entirely eliminates the burden at the implementation stage.

## Predictive modeling for Lissajous search pattern design

### Data and methodology

As Fig. 14 shows, under most conditions there are several ranges of nearly-optimal frequency ratios for which search performance varies only minimally. In fact, most values of $r_\omega$ result in moderately acceptable performance as compared with the assorted outlier “spikes”. Thus, an alternate approach to Lissajous search pattern design may ask not the question, “What frequency ratio is optimal?” but rather “What range(s) of frequency ratios should be avoided?” When a rigorously exact globally optimal solution is not required, data-driven approaches can provide an adequate solution much faster than pure simulation alone. This is afforded by the fact that such a predictive modeling approach bears the computational burden of simulation in offline training during model generation rather than online querying.

The frequency ratio $r_\omega$ is an explanatory feature which only partially explains the response variable $E[T_\alpha ]$, but there are several other features (subject to the assumptions of section “Expected distance to a uniform random target”) that also affect the response. In particular, these include: (1) the size of the agent’s FOV as a percentage of the area of the search space, (2) the time constant $\tau$ from Eq. 35, and (3) the certainty threshold $\alpha$ from Eq. 37. To build the predictive models, 86,999 simulations were executed with each explanatory variable being randomly generated and the response variable recorded. Ten percent of these trials were retained as a testing set, while the rest were used to train predictive models. Table 3 summarizes the statistics of the simulations. The range of values for %FOV, $\alpha$, and $\tau$ were chosen to roughly represent realistic scenarios.

**Table 3:** Training set summary statistics.

	$E[T_\alpha ]$	$r_\omega$	%FOV	$\alpha$	$\tau$
Min.	67.2	0.0025	0.0010	0.5000	1.00
1st Qu.	585.0	0.2551	0.0142	0.6213	10.53
Median	967.2	0.5043	0.0263	0.7444	20.03
Mean	1637.4	0.5025	0.0262	0.7459	20.22
3rd Qu.	1834.0	0.7536	0.0382	0.8702	29.83
Max	19904.0	0.9999	0.0499	1.0000	40.00

### Modeling

**Figure 15:**
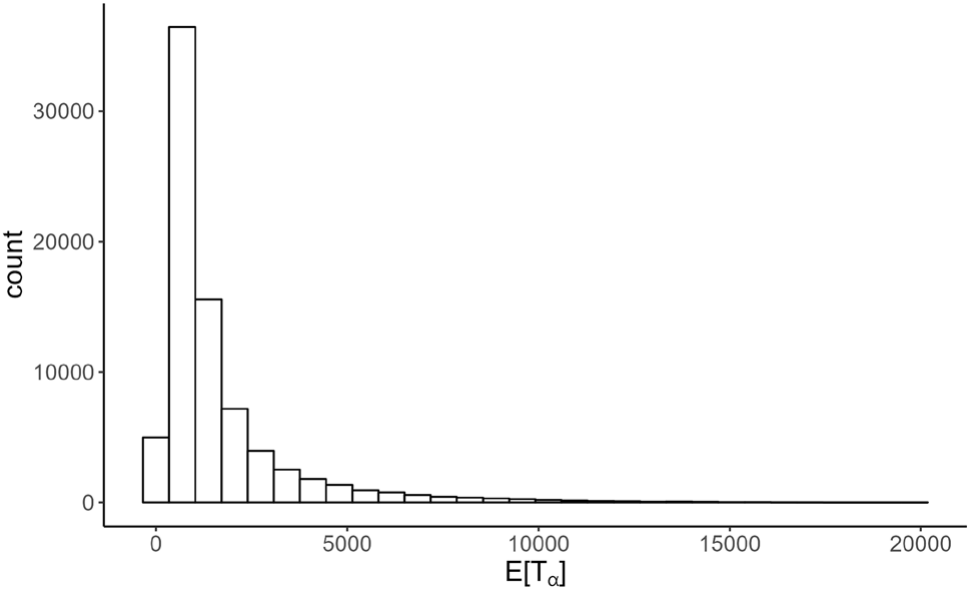
Distribution of the independent response variable $E[T_\alpha ]$.

To begin, a basic linear regression was used to predict the expected mission completion time from the four simulation input variables. After building and testing the linear model, only $35.74\%$ ($R^2 = 0.3574$) of the variation in the data was explained, and the root-mean-squared-error (RMSE) in predicting the test set was 1573.542 as shown in Table 4. Including the interaction terms between each of the four explanatory features only marginally improved model performance, increasing the $R^2$ to 0.3929 and decreasing the RMSE to 1519.414, still a poor model. However, linear regression inherently assumes a normal distribution of the response, and as Fig. 15 shows, the distribution of $E[T_\alpha ]$ is much more resemblant of a gamma distribution.

Taking this clue, the linear model was generalized to use a logarithmic link function and assume an underlying gamma distribution of the response. The resulting gamma regression model provided improved results with a pseudo-$R^2$ ($R_\text {L}^2$) of 0.5609 and RMSE of 1437.222. The $R_\text {L}^2$ measure relies on a likelihood ratio, hence the “L” subscript, and is calculated by

38 $$\begin{aligned} R_\text {L}^2 = \frac{D_0 - D_k}{D_0}, \end{aligned}$$

where $D_0$ is the model’s null deviance and $D_k$ is the residual deviance with *k* degrees of freedom^29^.

**Figure 16:**
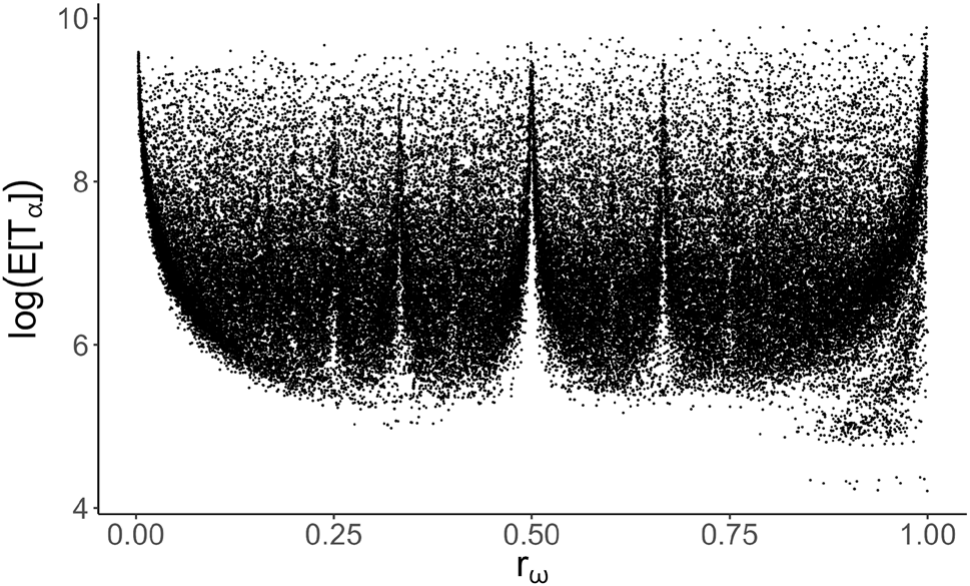
Strongly nonlinear relationship between frequency ratio $r_\omega$ and the logarithm of the response $E[T_\alpha ]$.

While gamma modeling is the most appropriate regression generalization for the problem at hand, basic regression is still fundamentally insufficient to adequately model the Lissajous simulation data. Traditional gamma modeling with a logarithmic link function assumes a linear relationship between the explanatory features and the logarithm of the response, but Fig. 16 shows clear evidence of a nonlinear relationship.

However, a generalized additive model (GAM) provides a more flexible method that can characterize nonlinear regression effects^30^. While a basic linear model assigns to each predictor an associated coefficient $\beta _i$:

39 $$\begin{aligned} E(Y|X_1,X_2,X_2,X_4,X_5)&= \beta _0 + \beta _1 X_1 + \beta _2 X_2 \nonumber \\ \&+ \beta _3 X_3 + \beta _4 X_4, \end{aligned}$$

a generalized additive model fits a non-parametric function $f_i$ (natural cubic splines in this case) to each predictor as shown in Eq. (40):

40 $$\begin{aligned} E(Y|X_1,X_2,X_2,X_4,X_5)&= \beta _0 + f_1(X_1) + f_2(X_2) \nonumber \\&+ f_3(X_3) + f_4(X_4). \end{aligned}$$

A GAM allows the modeling of nonlinear effects while still leveraging an underlying gamma distribution to achieve better results than either previous technique. A GAM using natural splines has an additional hyperparameter for each non-parametric function it fits, realized as the number of “knots” for each spline. Using five knots for each function yields an $R_\text {L}^2$ of 0.7052 and an RMSE of 1270.333. Increasing the number of knots can increase $R_\text {L}^2$, apparently improving the model, but this also increases the RMSE, which is a sign of over-fitting. Thus, five knots were heuristically chosen for the results delivered here. A summary of the applied statistial models is given in Table 4.

**Table 4:** Model performance metrics.

Model	$R_\text {(L)}^2$	RMSE
Linear Regression	0.3574	1573.542
Linear Regression (w/interactions)	0.3929	1519.414
Gamma Regression	0.5609	1437.222
Generalized Additive Model	0.7052	1270.333

### Discussion and future work

While even the GAM is still unlikely to deliver optimal LP parameters as reliably as an entirely numerical approach, this investigation shows that predictive modeling for path design has promise for the future. Follow-on work will build on these findings to develop a model that rivals other methods in accuracy and delivers near-optimal Lissajous flight paths more efficiently. A first step for future work is to generate more training data. While nearly 80, 000 training observations is no insignificant amount for most low-dimensional linear modeling problems, even a modest resolution of each parameter dimension in the problem at hand yields almost 300 trillion required data points to fully fill out the feature space. More data would also benefit machine learning models outside the scope of this paper (e.g. neural networks, random forests, or ensemble methods) which could be a good fit for the nonlinear nature of this problem. Another possibility for handling linearity issues could be a form of complex feature engineering, or perhaps more complex simulations that account for more information and variables that are predictive of $E[T_\alpha ]$.

## Conclusions and future applications

Using Lissajous curves as deterministic search patterns for aerial vehicles is a venture which shows promise, and thus designing optimal Lissajous paths is an important task. While many interesting and perhaps unintuitive findings can be gleaned through an analytical exploration of the problem, it is a problem so complex that a full solution is simply unattainable through purely analytical means. A numerical approach circumvents this intractability, providing a means of designing effective Lissajous search paths, but this comes at the cost of a high computational burden. Predictive modeling shows promise as an alternative, offering quick on-the-fly LP design, but a more accurate data-driven model is still required before the approach can be applied to real aerial systems.

When deterministic search patterns are sought, the need for immediate online path generation is generally lower than the need for a pattern that quickly and successfully completes a mission. Thus, the next phase of work will focus on improving the fidelity of the numerical simulator in order to better handle a more diverse set of missions. A satisfactory end-state of this research would be a multi-purpose software platform, driven by simulation and machine learning, which provides the user with an optimal path for any specified aerial search scenario. Such a platform would appropriately account for any contextual information provided by the user to generate waypoints for a manned or unmanned aircraft.

### Supplementary Information

Supplementary Information.

## Data Availability

All data generated or analyzed during this study are included in this published article’s supplementary information files or can be obtained by request from the corresponding author.

## Appendix

### A. Generalized discrete Lissajous formulation

Deriving the generalized recursive model for a Lissajous curve is accomplished by repeatedly transforming and de-transforming the state vector at each time step. The linear transformation $\mathbf {\mathcal {R}}{\textbf{x}} + \overline{{\textbf{x}}}$ rotates and shifts the vector ${\textbf{x}}$. The initial state vector given by Eq. (12) is first transformed in this way, mapping the first coordinate in the Lissajous curve to the desired location in the plane. Before the state propagation of Eq. (10) can be applied to obtain ${\textbf{x}}_1$, the state must first be de-transformed according to ${\textbf{x}}_0' = \mathbf {\mathcal {R}}^{-1}({\textbf{x}}_0 - \overline{{\textbf{x}}})$. After applying Eq. (10), the state vector is again transformed to deliver the desired rotation and shift: ${\textbf{x}}_1 = \mathbf {\mathcal {R}}({\textbf{A}}{\textbf{x}}_0') + \overline{{\textbf{x}}}$. Combining these operations yields the following expression:

41 $$\begin{aligned} {\textbf{x}}_1 = \mathbf {\mathcal {R}}({\textbf{A}}\mathbf {\mathcal {R}}^{-1}({\textbf{x}}_0 - \overline{{\textbf{x}}})) + \overline{{\textbf{x}}}. \end{aligned}$$

Noting that the rotation matrix is orthogonal and generalizing the time steps, this becomes:

42 $$\begin{aligned} {\textbf{x}}_k = \mathbf {\mathcal {R}}{\textbf{A}}\mathbf {\mathcal {R}}^{\top }{\textbf{x}}_{k-1} - \mathbf {\mathcal {R}}{\textbf{A}}\mathbf {\mathcal {R}}^{\top }\overline{{\textbf{x}}} + \overline{{\textbf{x}}}, \end{aligned}$$

or

43 $$\begin{aligned} {\textbf{x}}_k = \mathbf {\mathcal {R}}{\textbf{A}}\mathbf {\mathcal {R}}^{\top }{\textbf{x}}_{k-1} + ({\textbf{I}} - \mathbf {\mathcal {R}}{\textbf{A}}\mathbf {\mathcal {R}}^{\top })\overline{{\textbf{x}}}. \end{aligned}$$

Equation (14) follows directly from this result.

### B. Evaluating the distance integral

Evaluating the inner integral of Eq. (23) gives:

44 $$\begin{aligned} \begin{aligned}{}&\frac{1}{3}\int _{-\tan ^{-1}{\big (\frac{1/2+y_S}{1/2-x_S}\big )}}^{\tan ^{-1}{\big (\frac{1/2-y_S}{1/2-x_S}\big )}} r^3|_{0}^{\big (\frac{1/2-x_S}{\cos {\theta }}\big )} d\theta \\&\quad = \frac{(1/2-x_S)^3}{3}\int _{-\tan ^{-1}{\big (\frac{1/2+y_S}{1/2-x_S}\big )}}^{\tan ^{-1}{\big (\frac{1/2-y_S}{1/2-x_S}\big )}} \frac{1}{\cos ^3{\theta }} d\theta . \end{aligned} \end{aligned}$$

The final integral gives:

45 $$\begin{aligned} \begin{aligned}{}&\frac{(1/2-x_S)^3}{6}\Big [\frac{\sin {\theta }}{\cos ^2{\theta }}+\ln {\big (\cos {(\theta /2)}+\sin {(\theta /2)}\big )} \\&\quad - \ln {\big (\cos {(\theta /2)}-\sin {(\theta /2)}\big )} \Big ]_{-\tan ^{-1}{\big (\frac{1/2+y_S}{1/2-x_S}\big )}}^{\tan ^{-1}{\big (\frac{1/2-y_S}{1/2-x_S}\big )}}. \end{aligned} \end{aligned}$$

After substitution, rearrangement, and algebraic/trigonometric simplifications, this becomes:

46 $$\begin{aligned} \begin{aligned}{}&\frac{(1/2-x_S)^3}{6}\Bigg [ \Big (\frac{1/2-y_S}{1/2-x_S}\Big )\sqrt{\Big (\frac{1/2-y_S}{1/2-x_S}\Big )^2 + 1} \\&\quad + \Big (\frac{1/2+y_S}{1/2-x_S}\Big )\sqrt{\Big (\frac{1/2+y_S}{1/2-x_S}\Big )^2 + 1} \\&\quad + 2\tanh ^{-1}{\Bigg (\frac{\sqrt{\Big (\frac{1/2-y_S}{1/2-x_S}\Big )^2 + 1}-1}{\Big (\frac{1/2-y_S}{1/2-x_S}\Big )}\Bigg )} \\&\quad + 2\tanh ^{-1}{\Bigg (\frac{\sqrt{\Big (\frac{1/2+y_S}{1/2-x_S}\Big )^2 + 1}-1}{\Big (\frac{1/2+y_S}{1/2-x_S}\Big )}\Bigg )}\Bigg ]. \end{aligned} \end{aligned}$$

This is obtained for each of the other three regions of integration by appropriately changing the integration limits. Once completed, the combined integral simplifies to the result given in Eq. (24).

### C. Average expected squared distance

Consider the expected value of the squared distance between a searcher and a target:

47 $$\begin{aligned} \begin{aligned} E[d^2(x_S,y_S)]&= \int _{\mathcal {D}} f_{X_T,Y_T}(x_T,y_T)d^2(x_S,y_S,x_T,y_T) dA \\&= \int _{-0.5}^{0.5}\int _{-0.5}^{0.5} \big ((x_S-x_T)^2+(y_S-y_T)^2\big ) dx_Tdy_T \\&= x_S^2 + y_S^2 + \frac{1}{6}. \end{aligned} \end{aligned}$$

Enacting the assumptions of section “Expected distance to a uniform random target”, this can be written for an LP:

48 $$\begin{aligned} E[d^2(x_S,y_S)] = \frac{1}{4}\cos ^2{(r_\omega \omega _{y}t)} + \frac{1}{4}\cos ^2{(\omega _{y}t)} + \frac{1}{6}. \end{aligned}$$

The average expected squared distance over a period of time *T* is then:

49 $$\begin{aligned} \begin{aligned} {\overline{D}}^2(r_\omega ,T)&= \frac{1}{T}\int _0^T \big (\frac{1}{4}\cos ^2{(r_\omega \omega _{y}t)} + \frac{1}{4}\cos ^2{(\omega _{y}t)} + \frac{1}{6}) dt \\&= \frac{1}{8}\big ({{\,\textrm{sinc}\,}}{(2r_\omega \omega _yT)} + {{\,\textrm{sinc}\,}}{(2\omega _yT)}\big ) + \frac{5}{12}. \end{aligned} \end{aligned}$$

It can then be shown without much difficulty that the average expected squared distance over a long time is

50 $$\begin{aligned} {\overline{D}}^2(r_\omega ) = \lim _{T \rightarrow \infty } {\overline{D}}^2(r_\omega ,T) = \frac{5}{12}, \end{aligned}$$

when the search domain is the unit square. It is worth noting that ${\overline{D}}(r_\omega ) \ne \sqrt{{\overline{D}}^2(r_\omega )}$ since the square root is not a linear operation. Indeed, $\sqrt{\frac{5}{12}} \ne 0.585$.

### D. Proof of the optimal symmetric linear approximation of the Pythagoream theorem

We seek a value for $\alpha$ which optimizes the approximation $\alpha \big (|x|+|y|\big )$ to $\sqrt{x^2+y^2}$. Since *x* and *y* should be interchangeable, let $\lambda = \frac{y}{x}$ such that $\lambda \in (0,1)$ and formulate an objective function as:

51 $$\begin{aligned} \begin{aligned} O&= \Big |\sqrt{x^2+y^2} - \alpha \big (|x|+|y|\big )\Big | \\&= \Big ||x|\sqrt{1+\lambda ^2} - \alpha |x|\big (1+\lambda \big )\Big |. \end{aligned} \end{aligned}$$

Clearly, this is minimized when $\sqrt{1+\lambda ^2} - \alpha \big (1+\lambda \big ) = 0$, or

52 $$\begin{aligned} \alpha = \frac{\sqrt{1+\lambda ^2}}{1+\lambda }. \end{aligned}$$

If the ratio of *x* and *y* is known, the optimizing value of $\alpha$ can be determined from the above. However, we wish to obtain $\alpha$ which minimizes the average *O* over the full range of $\lambda$. This average value is

53 $$\begin{aligned} \begin{aligned} {\overline{\alpha }}&= \int _0^1 \frac{\sqrt{1+\lambda ^2}}{1+\lambda } d\lambda \\&= -1 + \sqrt{2} - \sinh ^{-1}{(1)} + \sqrt{2}\tanh ^{-1}{\Big (\frac{1}{\sqrt{2}}\Big )} \\&\approx 0.77929. \end{aligned} \end{aligned}$$

The percent error in this estimate is given by:

54 $$\begin{aligned} \epsilon = \frac{O}{\sqrt{x^2+y^2}} = \frac{\Big |\sqrt{1+\lambda ^2} - {\overline{\alpha }}(1+\lambda )\Big |}{\sqrt{1+\lambda ^2}}. \end{aligned}$$

Over the range of possible $\lambda$, $\epsilon$ takes on a maximum value when $\lambda = 0$:

55 $$\begin{aligned} \epsilon _{max} = \Big |1 - {\overline{\alpha }}\Big | \approx 22.071\%. \end{aligned}$$

## Abbreviations

UAS: Unmanned aerial systems
UAV: Unmanned aerial vehicles
2D: Two-dimensional
LP: Lissajous pattern
CS: Circle search
SS: Sector search
PTS: Parallel track search
ES: Expanding square
FOV: Field of view
CDF: Cumulative density function
AUC: Area under the CDF
AAC: Area above the CDF
RMSE: Root-mean-squared-error
GAM: Generalized additive model
